# Reducing classroom sedentary behaviour: a scoping review of interventions and student involvement

**DOI:** 10.1093/heapro/daaf167

**Published:** 2025-10-15

**Authors:** Sofia Dinkelspiel Ekman, Monika Nair, N Viktor Gredin, Eva-Carin Lindgren

**Affiliations:** School of Health and Welfare, Halmstad University, Box 823, SE-301 18 Halmstad, Sweden; School of Health and Welfare, Halmstad University, Box 823, SE-301 18 Halmstad, Sweden; School of Health and Welfare, Halmstad University, Box 823, SE-301 18 Halmstad, Sweden; School of Health and Welfare, Halmstad University, Box 823, SE-301 18 Halmstad, Sweden

**Keywords:** classroom interventions, co-design, empowerment, sedentary, stand-biased desks, standing desks

## Abstract

Sedentary behaviour is increasingly common and is becoming a challenge to maintain the young population healthy. This scoping review aims to identify the existing literature on strategies for altering the classroom design to reduce sedentary behaviour in classroom settings during lessons and the impact of interventions. Three electronic databases (ERIC, PsycInfo, and Scopus) were searched for relevant literature. A total of 40 eligible articles from different countries published between January 2003 and April 2024 were included in the review, which was carried out to identify interventions to prevent sedentary behaviour by redesigning the classroom. The methodology was conducted by the framework presented by Arksey and O'Malley and is reported following the PRISMA-ScR guidelines. The findings reveal various approaches to preventing sedentary behaviour through active desks (e.g. standing desks, sit-to-stand desks, bicycling desks, and stability balls). Most studies focused on stand-biased desks, targeting students (9–12 years old) and were primarily from the USA. Since standing was voluntary, only minor bouts of standing and movement were typically achieved. Only a few studies included partial co-design elements. The thematic analysis identifies the impact of the different interventions. Five themes were found: impact on physical activity, classroom behaviour, cognitive performance, physical and/or mental well-being and challenges. The findings suggest that standing at standing desks while studying does not pose significant physical or mental challenges for students, although some physical fatigue was observed after prolonged standing up. However, a key gap was the absence of co-design in the intervention process.

**Trial registration:** The scoping review protocol was not pre-registered.

Contribution to Health Promotion

- Promoting an active lifestyle by reducing sedentary behaviour in educational settings for children and adolescents.
- Addressing easy access environmental factors by using the classroom to support healthier behaviour.
- Valuable insights into effective strategies of existing interventions aimed at reducing sedentary behaviour for policymakers and educators.
- Raising the importance about a more holistic approach through inclusivity and sustainability where the end-users are involved in the process through co-design.

## INTRODUCTION

People in high-income countries spend most of their waking hours sedentary, today more than ever, and adolescents more than average (Carson *et al*. 2016, Kontostoli *et al*. 2021). Sedentary behaviour is generally defined as any waking behaviour with an energy expenditure of ≤1.5 metabolic equivalents (METs) in a lying, reclining or sitting posture (Tremblay *et al*. 2017). As sedentary behaviour increases, health outcomes progressively worsen (Tremblay *et al*. 2011). Sedentary behaviour is linked to a variety of adverse health effects as it induces physiological changes such as skeletal muscle atrophy, impaired glucose uptake, endothelial dysfunction, which in turn fosters the metabolic environment necessary for the onset and progression of chronic diseases like type 2 diabetes, cardiovascular disease, and non-alcoholic fatty liver disease (Murtagh *et al*. 2016, Kopp 2019, Schnurr *et al*. 2020, Pinto *et al*. 2023). Given the long-term health risks associated with prolonged sedentary behaviour, early intervention is essential. Targeting children—whose habits and environments are still developing—may be more impactful than focusing on adults, as behavioural patterns formed during childhood tend to persist into later life (Kuzik *et al*. 2022). This positions children as a key group for preventive efforts aiming to shift sedentary norms.

A natural arena for researching sedentary behaviour in children is in school. School-aged children spend an essential part of their waking time in school, where sedentary behaviour is predominant (Kuzik *et al*. 2022). Given this, researching reducing sedentary behaviour within schools is both logical and widely practised (Álvarez-Bueno *et al*. 2017, Andermo *et al*. 2020). Beyond physical health, excessive sedentary time during lessons is linked to reduced classroom engagement, lower executive function, and increased cognitive fatigue (Donnelly *et al*. 2016, Bellacicco *et al*. 2025). As such, the classroom presents a strategic setting not only for learning but for promoting holistic student health—supporting both educational outcomes and lifelong healthy behaviours.

Classroom designs have historically been inspired by industrialization and the efficient seating of workers in assembly lines, where control and discipline have been essential characteristics (Serafini 2002). However, shifts in focus within educational philosophy, where more student-centred learning has grown more popular, are challenging this norm. One such approach to designing educational environments is co-design (Bowler *et al*. 2021), a participatory, iterative process where the students and teachers collaborate in the redesign of the classroom. It involves shared decision-making, and joint development of ideas to ensure that the designs are more inclusive, effective and relevant. Interventions may benefit from co-design approaches that begin with students and teachers to ensure contextual relevance and then extend to researchers and system-level stakeholders to align feasibility with evidence-based practise. Engaging both students and teachers as collaborative partners is essential for effective and context-sensitive intervention design (Bernal *et al*. 2023).

This scoping review aims to map existing research on classroom design interventions intended to reduce sedentary behaviour in school settings, with particular attention to the role of co-design (Bowler *et al*. 2021, Olsen 2023). While several reviews have examined classroom-based modifications to reduce sedentary behaviour (Minges *et al*. 2016, Sherry *et al*. 2016, Guirado *et al*. 2021, Sánchez-López *et al*. 2025), these reviews have not systematically examined the extent of participatory design approaches or student involvement in intervention development. This represents a notable gap, as our preliminary investigation suggested that co-design terminology in this literature might be inconsistent or absent entirely, necessitating a comprehensive search strategy that captured all classroom design interventions regardless of their stated design methodology. Co-design may offer unique value in tailoring interventions to students’ needs, increasing relevance, and promoting sustainable behavioural change. Involving students and educators in the design process may enhance the acceptability, effectiveness, and contextual fit of interventions—factors critical to long-term impact in educational settings.

The following three research questions shall guide the study:

1. What are the characteristics of interventions targeting classroom design to reduce sedentary behaviour during lessons?
2. To what extent are students involved in the co-design of these interventions?
3. What impacts do classroom design interventions have on reducing sedentary behaviour?

## MATERIALS AND METHODS

### Data collection

This scoping review was conducted by the framework presented by Arksey and O'Malley (2005) and follows the PRISMA guidelines for scoping reviews (PRISMA-ScR; Tricco *et al*. 2018) to enhance rigour and transparency. The selection of a scoping review methodology is informed by the heterogeneity of the available evidence and the intention to map key concepts and identify gaps in this broad field.

The inclusion criteria were: (i) Children aged 6–16 years, corresponding to compulsory schooling in Sweden; this age range served as a reference point only; the reviewed studies were conducted internationally. (ii) Experimental or intervention study design. (iii) Focus on modifications to the physical classroom environment (furniture, layout, and equipment) aimed at influencing student movement and posture during regular instructional time. (iv) Targeting physical activity (PA), movement, and sedentary behaviour. (v) Peer-reviewed articles published in English. (vi) Studies dated between January 2004 and April 2024.

The exclusion criteria were: (i) preschool or university students, (ii) research focusing solely on adult or non-school populations, (iii) studies that do not measure or discuss PA or movement, (iv) editorials and opinion pieces, (v) study protocols, surveys, obesity studies, health or nutrition studies, unless PA or sedentary behaviour related to classroom design was a primary aim, PA breaks in the classroom or at breaks, as well as teacher-led PA in the classroom.

The criteria were chosen to focus specifically on school-aged children (6–16 years), aligning with the period of compulsory education in Sweden, during which classroom-based sedentary time is most structured and sustained. This age group also represents a key developmental window for establishing lifelong movement habits. We focused on intervention or experimental studies in classroom settings to identify strategies that directly modify the learning environment itself, rather than behavioural programmes or teacher-led activity breaks. Excluding studies on preschoolers, university students, or non-school populations helped maintain contextual relevance. Limiting the search to peer-reviewed articles published in English from 2004 onward ensured scientific rigour and relevance to contemporary classroom practises and educational policies.

The search string used in ProQuest for ERIC and PsycInfo was

NOFT(movement OR ‘physical activity’ OR sedentar* OR sitting OR standing OR stand) AND NOFT(classroom) AND NOFT(intervention* OR experiment*) AND RTYPE(‘Article’) AND STYPE(‘Scholarly Journals’) AND LA(eng) AND YR(2003–2024) AND PEER(yes)

And for Scopus:

TITLE-ABS-KEY (movement OR ‘physical activity’ OR sedentar* OR sitting OR standing OR stand) AND TITLE-ABS-KEY (classroom) AND TITLE-ABS-KEY (intervention* OR experiment*) AND DOCTYPE(ar OR er OR re) AND LANGUAGE(english) AND PUBYEAR AFT 2002

The search strategy deliberately excluded co-design terminology (e.g. ‘co-design’, ‘participatory design’, and ‘user-centred design’) to avoid selection bias towards studies that explicitly used these terms. This approach enabled comprehensive mapping of all classroom design interventions targeting sedentary behaviour, allowing systematic examination of student involvement across the entire landscape rather than only within studies that self-identified as participatory. This methodological choice was validated by our findings, which revealed that no studies in the broader literature employed explicit co-design approaches.

### Data analysis

**Figure 1.**
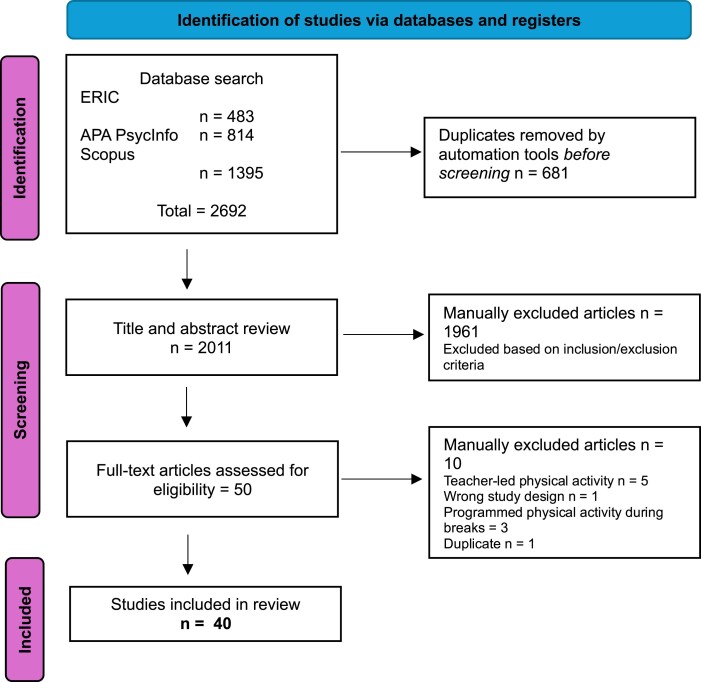
PRISMA 2020 flow diagram adapted from Page *et al*. (2021).

The Rayyan.ai tool was used to review all the articles; for the first screening, a title and abstract review of the 2011 articles was carried out, a procedure that guaranteed the revision of each paper by at least three authors. Title and abstract screening was conducted independently by two reviewers. Discrepancies were resolved through discussion with a third reviewer, ensuring consistency and transparency in selection. The 1961 articles were manually excluded based on the inclusion and exclusion criteria. A second screening with full-text assessment was carried out by at least two authors of the remaining 50 articles, where 10 more articles were excluded based on the same criteria. Full-text screening followed the same procedure as with the title and abstract screening, with all disagreements resolved through group discussion in the research team. A final selection of 40 articles was agreed upon by the four authors to become the foundation on which this review was written (Fig. 1).

To address research questions 1 and 2—(1) What are the characteristics of interventions targeting classroom design to reduce sedentary behaviour during lessons? and (2) To what extent are students involved in the co-design of these interventions?—the selected 40 articles were first reviewed by one author to gain an overall understanding of research characteristics related to classroom design interventions aiming to reduce sedentary behaviour during lessons. Next, the articles’ research characteristics were compiled into a table including author, year of publication, country, study aims, participants, and study design (Table 1). All collaborations between the research team and end-users to respond to the second research question of to what degree the students are involved in co-designing classrooms to prevent sedentary behaviour during lessons were examined. To answer the third research question—3. What impacts do classroom design interventions have on reducing sedentary behaviour?—an inductive thematic analysis was conducted. This thematic analysis followed the steps defined by Braun and Clarke (2006). First, the articles were read several times by one author to obtain an overall impression. Second, identifying and marking important data characteristics to generate initial codes responding to the aim was carried out by the same author. Third, all the authors discussed, compared, and organized the codes based on similarities and differences and then grouped them into tentative themes. Fourth, all the authors discussed and reviewed the tentative themes several times and adjustments were made by checking the data for eligibility. Frequent debriefing sessions were done to ensure credibility (Lincoln and Guba 1985). Fifth, the final themes and sub-themes were defined and named. Sixth, the analysis was written, showing themes through data extracts (Table 2).

**Table 1.** The studies’ characteristics.

Author, year, and country of publication	Study aim	Study design and duration	Co-design	Participants, sample size, CG	Classroom design
Aminian *et al*. (2015, New Zealand)	Evaluate whether height-adjustable workstations in dynamic classrooms reduce sitting and increase standing	Mixed method, controlled trial: 9-week intervention followed by 13-week continuation. Interview + FGI, Musculoskeletal + Swan questionnaires	No	Mean/SD 9.8 yoa. IG = 18, CG = 8	Height-adjustable desks with alternative seating (e.g. stability balls, bean bags)
Aspiranti *et al*. (2023, USA)	Examine if movement-based desks and alternative seating can boost PA and academic outcomes in children with disabilities	Quantitative, pilot feasibility study with an 8-week crossover design	Teacher brainstorming on rule implementation	Fourth to fifth grade (9–11 years) mean/SD not reported. IG = 14 and seventh to eighth grade (12–14 years), IG = 21	Action workstations (pedal, spin, balance) with flexible seating and rotatione
Benden *et al.* (2012, USA)	Examine whether stand-biased desks could increase EE and reduce obesity risks in children	Quantitative, within-subject intervention with two consecutive 5-month trials	No	6–8 yoa, mean/SD not reported. IG = 9	Standing height desks
Benden *et al.* (2014, USA)	Investigate if standing desks increase energy expenditure and PA to combat childhood obesity	Quantitative, quasi-RCT intervention. One-week sampling in fall and spring	No	Second to fourth grade (mean/SD 8.5). IG = 202, CG = 135	Stand-adjustable desks
Blake *et al.* (2012, USA)	Examine standing desks as a way to increase PA in classrooms without using extra instructional time	Mixed method, 1-year quasi-experimental pilot study	No	6–7 years old (mean/SD not reported) five classes, IG = 2 classes, CG = 2 classes, one class with within-class comparison	Stand/sit workstations and stools
Brittin *et al.* (2017, USA)	Examine if a movement-promoting school environment can reduce SB and increase PA in elementary students	Quantitative, natural quasi-experiment for 14 months	No	Third graders (8–9 years); follow-up with fifth graders (10–11 years) mean/SD not reported. IG = 21 CG = 20	Height-adjustable chairs, sit-to-stand tables, stools, soft seating, and beanbags
Chen *et al.* (2021, UK)	Assess sit-stand desks’ impact on reducing SB and increasing light PA	Quantitative, pilot trial, a two-arm cluster RCT, 4, 5 months	No	Mean/SD 9.3 yoa, (eight schools). IG = 86 CG = 90	Sit-stand desks with classroom rotation system and teacher encouragement to stand
Clemes *et al.* (2016, UK and Australia)	Investigate whether introducing sit-to-stand desks in classrooms effectively reduces children’s sitting time	Quantitative, pilot controlled trials, 9–10 weeks	No	9–10 yoa, 10–11 yoa, mean/SD not reported. IG = 16, CG = 14 in the UK and IG = 24, CG = 20 in Australia.	Sit-stand desks in the UK and Australia with group rotation and 1-h exposure
Clemes *et al*. (2020, USA)	Evaluate the feasibility and acceptability of a sit-stand desk intervention in primary schools, and its potential to reduce children's sitting time	Mixed method, a two-armed pilot cluster RCT. 4, 5 months	Participatory feedback, but not co-design	9–10 yoa, mean/SD not reported. IG = 86, CG = 90	Sit-stand desks with daily rotation and teacher encouragement to stand
Ee *et al.* (2018, Australia)	Evaluate whether standing desks in classrooms can increase standing time, reduce sitting time, and decrease musculoskeletal discomfort in children	Quantitative, within-subjects crossover study design, 21 + 21 days	No	10–11 yoa, mean/SD not reported. IG = 47 male students	Height-adjustable desks with fidget bars, allowing students to swing their non-supporting leg while standing. Used in a crossover design
Erwin *et al.* (2016, USA)	Evaluate whether stability balls increase PA and positively influence student behaviour compared with regular chairs	Quantitative, quasi-experimental, 10 school days	No	Fourth graders (−10 yoa) mean/SD not reported. IG = 15, CG = 15 (on PA), IG = 2, CG = 2 (on/off-task)	Stability balls used as seating during class
Erwin *et al*. (2018, USA)	Assess the feasibility of sit-to-stand desks in promoting standing, reducing misbehaviour, and exploring student and teacher feedback on classroom dynamics and learning behaviours	Mixed method, feasibility study during six different class periods per day for 45 min each, 5 weeks	No	Sixth to eighth graders (11–14 yoa) mean/SD not reported. IG only = 18 in sixth grade, IG only = 14 in seventh grade, IG only = 16 in eighth grade	Sit-stand desks used voluntarily; desks observed after 9-week exposure
Fedewa *et al.* (2018, USA)	Investigate the potential of bicycle workstations to increase low-intensity PA in high school students without negatively affecting classroom behaviour	Mixed method, quasi-experimental within-subject design. Pilot study, 4 months	No	Mean/SD 1 606 yoa. IG = 80	Bicycle workstations used in rotation with regular desks
Fedewa *et al*. (2021, USA)	Evaluate the feasibility of using desk cycles in classrooms to increase PA, and assess their impact on on-task behaviour	Mixed method, feasibility study. Two weeks data collection for each group (totally 60 school days)	No	Fifth grade students (10–11 yoa), mean/SD not reported. IG = 15, CG = 13	Desk cycles: used at needed by students
Fedewa *et al*. (2017, USA)	Investigate whether stationary bicycle desks (FitDesk) can increase PA levels, particularly MVPA, in adolescents during school hours	Mixed method, quasi-experimental design over an academic year	No	14–18 yoa (Grades 8–12), mean/SD not reported. IG = 11, CG = 6	FitDesks (stationary bikes) rotated among students during class time
Hartikainen *et al.* (2022, Finland)	Investigate whether open and flexible classroom designs can reduce SB in students by promoting shorter sedentary bouts and increasing sit-to-stand transitions	Quantitative, cross-sectional study, 1 week	No	Third and fifth grade students, mean/SD not reported. IG = 59, CG = 132	Open-plan classrooms with mobile furniture and no assigned seating
Hartikainen *et al*. (2021, Finland)	Assess whether open and flexible classroom designs, introduced after the Finnish curriculum reform, can reduce SB and increase PA	Quantitative, cross-sectional case study. One week × 2 (during two separate academic years)	No	9 and 11 yoa, third grade, mean/SD not reported. IG = 41 and fifth grade IG = 42, before renovation. Third grade IG = 19 and fifth grade IG = 28 after renovation	Traditional classrooms converted to open learning spaces with zones
Hinckson *et al*. (2013, New Zealand)	Evaluate the acceptability and impact of standing workstations in elementary classrooms	Mixed method, controlled trial. Feasibility study, March–May 2012	No	Third and fourth grades, mean/SD 10 yoa, IG = 23, CG = 7	Standing desks at multiple heights with flexible seating (exercise balls, bean balls, and maths) for rest
Hulac *et al*. (2022, USA)	Investigate the effectiveness of stability ball seating on improving student on-task behaviour	Mixed method, experimental design: within-subjects repeated measures. 13 days	No	Third grade students, mean/SD not reported. IG = 24	Seating conditions alternated between chairs, stability balls, or student choice
Koepp *et al.* (2012, USA)	Evaluate the impact of standing desks on classroom performance, behaviour, in-class PA, and body mass index in students	Quantitative, pilot intervention study with a pretest–posttest design study. Pedometers, classroom behaviour was assessed by teacher observations. One year.	No	Sixth grade (mean/SD 11.3 yoa). IG = 8	Standing desks customized to elbow height
Kidokoro *et al.* (2019, Japan)	Evaluate the impact of height-adjustable standing desks on reducing SB and increasing PA among primary school children	Quantitative data. A quasi-experimental study with a controlled before-and-after design, 6 months	No	11–12 yoa, mean/SD not reported. IG = 18, CG = 20	Height-adjustable desks with optional seating retained
Mead and Scibora (2016, USA)	Assess whether different types of physical exercise during math instruction can improve standardized math test scores in sixth graders	Quantitative, quasi-experimental design. No random assignment as each preexisting class was assigned a different intervention. One academic year (2012–2013)	No	Three sixth-grade classes (11–12 yoa, mean/SD not reported). IG = 29, CG = 23	Stability balls used during a set math period
Mehta *et al.* (2015, USA)	Determine the neurocognitive benefits, i.e. improvements in executive functioning and working memory, of stand-biased desks and explore any associated changes in frontal brain function	Quantitative, longitudinal quasi-experimental pilot study with repeated measures (within-subject design). One academic school year	No	Mean/SD 14.3 yoa, IG = 34 freshman high school students	Stand-biased desks
Metz *et al*. (2020, USA)	Examine the effects of ball-chair seating on legibility, student behaviour, and classroom productivity	Quantitative, quasi-experimental study with ABAB within-subjects design. One-year experimental pilot study, 18 + 16 study days	No	First grade students over two consecutive years, mean/SD not reported. IG = 11 Year 1, IG = 19 Year 2	Ball-chair seating
Parrish *et al*. (2018, Australia)	Evaluate the feasibility, acceptability, and potential efficacy of a school-based intervention to reduce adolescent sitting time during the school day	Quantitative, two-arm parallel-group RCT. Quantitative data (surveys and ActivPAL3), 5 months	No	13–16 yoa, mean/SD not reported. IG = 43, CG = 43	Stand-biased desks with portable whiteboards and standing reminders. Rotation routine
Parry *et al.* (2019, Australia)	Assess the sit-stand behaviour, waking sedentary time and PA, and musculoskeletal discomfort following the provision of standing desks into a Grade 4 classroom	Quantitative, repeated-measures crossover design study. One full school year (measurement at start and end of school year)	No	Fourth grade (9–10 yoa, mean/SD not reported), only boys. IG = 23	Two standing desks for half of the classroom. The standing desks had a ‘fidget bar’ installed which allowed students to swing their non-supporting legs while standing
Polo-Recuero *et al.* (2023, Spain)	Assess the effects of a classroom-based PA programme, using bike desks, on academic and physical performance in adolescents	Quantitative, RCT with a pretest–posttest design, 10 weeks during their Spanish-language arts lesson	No	14–15 yoa, mean/SD not reported. IG = 28, CG = 27	Stationary bikes that integrate with a desk workspace
Schwenke and Coenen (2022, Germany)	Exploring the effectiveness of sit-stand tables in creating a more active learning environment	Mixed-method study with a quasi-experimental design. Move 3 accelerometer and qualitative interviews with teachers, 5 days	No	7–10 yoa and 11–13 yoa, mean/SD not reported. IG = 22, CG = 22. 13 teachers	Adjustable sit-stand tables with optional sitting
Sherry *et al.* (2020, UK)	Address the potential benefits and challenges of introducing standing desks in classrooms, particularly in deprived settings, to reduce SB and explore effects on health and cognitive outcomes	Pilot controlled trial. Mixed-method approach with a quasi-experimental design, 8 months	No	Year 5 (UK) (9–10 yoa, mean/SD not reported). IG = 22, CG = 27	Sit-stand desks with 5.5-h exposure and daily standing periods
Silva *et al*. (2018, Portugal)	Explore the effectiveness of a standing desk intervention in reducing SB and increasing PA among sixth-grade students	Mixed method, cluster non-RCT, 16 weeks	Partially (evaluation during intervention)	11–13 yoa, mean/SD not reported. IG = 22, CG = 27	Standing desks
Silva *et al*. (2019, Portugal)	Examine the accuracy and agreement between the GT3X accelerometer and the ActivPAL inclinometer in measuring SB among adolescents, particularly in detecting changes resulting from a classroom standing desk intervention	Quantitative, secondary data from Silva 2018 study, 16 weeks	No	11–13 yoa, mean/SD not reported. IG = 22, CG = 27	Standing desks
Silva *et al*. (2022, Portugal)	Explore whether replacing traditional classroom desks with standing desks can enhance cognitive function and academic achievement in sixth-grade students	Quantitative method, a controlled trial, secondary data from Silva 2018 study, 16 weeks	Partially (evaluation during intervention)	11–13 yoa, mean/SD not reported. IG = 22, CG = 27	Standing desks
Sprengeler *et al.* (2020, Germany)	Investigate whether providing standing desks in classrooms can reduce sitting time and increase standing and stepping time among primary school children, while also examining if these effects vary based on children's physical fitness levels	Quantitative, case crossover study, January–March 2018	No	Third-grade primary school, mean/SD not reported. IG = 18, CG = 18	Standing desks randomly assigned to half of students
Spring *et al.* (2023, USA)	Investigate the impact of an under-the-desk band on middle school students’ PA levels and academic engagement	Quantitative, quasi-experimental study with a non-randomized, controlled design, 14 weeks	No	Two sixth-grade English classes (11–12 yoa, mean/SD not reported). IG = 13 in Class A (PA analysis), and IG = 6 in Class B (the academic engagement analysis).	Under-the-desk fidget bands
Sudholz *et al*. (2023, Australia)	Evaluate the effectiveness of height-adjustable desks combined with prompts to reduce prolonged sitting time in classrooms and to explore the social and motivational factors that influence adolescents’ behaviour in breaking up sitting time	Quantitative, quasi-experimental study, 17 weeks	No	14–15 yoa, mean/SD not reported. IG = 55, CG = 50	Height-adjustable desks with visual prompts and behaviour nudges
Swartz *et al.* (2019, USA)	Evaluate whether stand-biased desks can reduce SB and increase PA among elementary and middle school students throughout the school year	Quantitative, crossover design study, 9 weeks × 4 or wGT3X-BT	No	Third grade, mean/SD not reported. IG = 22, fourth IG = 36, and sixth grade IG = 41	Stand-biased desks and height-matched stool for 50% of students; others used traditional seating
Torbeyns *et al.* (2017, Belgium)	Investigate whether using bike desks in the classroom could enhance adolescents’ physical health, energy expenditure, cognitive performance, brain functioning, and academic performance	Quantitative, a RCT with a pretest–posttest design, 15 weeks	No	Mean/SD 14.3 yoa. IG = 21, CG = 23	Cycling desks used 4 h/week at preferred intensity
Verloigne *et al*. (2018, Belgium)	Assess the effectiveness of standing desks in reducing sitting time and altering sitting-related behaviours among pupils in primary and secondary schools, and to evaluate the implementation process	Mixed method, FG interviews for future studies, cluster RCT, 6 months	No	10–11 yoa and 15–16 yoa, mean/SD not reported. Primary students IG = 10, CG = 8, secondary students IG = 10, CG = 8	Standing desks rotated every 25 min; motivational posters displayed
Wallace *et al.* (2022, USA)	Investigate the potential cognitive benefits of stand-biased desks in school-aged children, exploring how these desks might influence cognitive performance and behaviour over the course of a school year	Quantitative, a within-classroom crossover design, 9 weeks × 2, during one school year	No	Third grade IG = 35, fourth grade IG = 23, and sixth grade IG = 41 (8–12 yoa, mean/SD not reported).	Stand-biased desks with height-matched stool and teacher encouragement for 50% of students; others used traditional seating
Wick *et al.* (2018, Switzerland)	Explore whether the use of standing desks in classrooms can improve cognitive function	Quantitative, quasi-experimental design with a non-RCT, 11 weeks	No	Mean/SD 10.8 yoa. IG = 19, CG = 19	Individual standing desks with anti-slip foot mats

CG, control group; IG, intervention group; RCT, randomized controlled trial; SB, sedentary behaviour; yoa, years of age.

**Table 2.** Themes, sub-themes, and codes.

Theme	Sub-theme	Code	Study references
Theme 1: Impact on physical activity (definition: tangible effects on the subject, caused by physical movement or activity ≥ 1.5 MET, objective measurements)	1.1: Increase/decrease in physical activity	Increase in steps/energy expenditure/activity levels measured by accelerometer devices; BodyBugg, ActivePal, ActivePal3, Actigraph GT3X, Sensewear mini armband, and Polar F4 (heart-rate monitor)	Clemes *et al.* (2016), Torbeyns *et al.* (2017), Fedewa *et al*. (2017), Fedewa *et al.* (2018), Benden *et al.* (2012), Kidokoro *et al.* (2019), Chen *et al.* (2021)
		Decrease in steps/PA, measured by accelerometer devices; Actigraph, Actigraph GTX3+	Erwin *et al.* (2016), Spring *et al.* (2023)
	1.2: Changes in posture	Decrease in sedentary time, measured by accelerometer devices; ActivePAL, 3, 3C, Actigraph GT3X, GT1M, GT9X, wGT3X+, wGT3X-BT	Silva *et al*. (2018), Aminian *et al*. (2015), Clemes *et al.* (2016), Blake *et al.* (2012), Hinckson *et al*. (2013), Fedewa *et al*. (2017), Wick *et al.* (2018), Kidokoro *et al.* (2019), Ee *et al.* (2018), Clemes *et al*. (2020), Brittin *et al.* (2017), Sudholz *et al*. (2023), Sherry *et al.* (2020), Chen *et al.* (2021)
		Increase in standing time and/or movement, measured by accelerometer devices; ActivePAL, BodyBugg, Actigraph, GT9X, GT3X, wGT3X-BT	Aminian *et al*. (2015), Blake *et al.* (2012), Hinckson *et al*. (2013), Erwin *et al*. (2018), Sherry *et al.* (2020), Wick *et al.* (2018), Ee *et al.* (2018), Chen *et al.* (2021)
		More sedentary as the school year progressed, accelerometer devices; Actigraph GT3X or wGT3X-BT	Swartz *et al.* (2019)
		Increase in sedentary time, accelerometer devices; Sensewear armbands, Actigraph GT3+	Spring *et al.* (2023), Benden *et al.* (2014)
	1.3: Divergent Outcomes on Impact on PA	ActivePAL*, students with higher fitness level, more active when standing	Sprengeler *et al.* (2020)
		Move 3 accelerometer, students with higher fitness level, more break-ups from sitting	Schwenke and Coenen (2022)
		IPAQ-SF (heart-rate monitors), female students with lower fitness, benefitted more	Polo-Recuero *et al.* (2023)
		Actigraph GT3X+, more sedentary students at baseline became less sedentary compared with traditional desks	Swartz *et al.* (2019)
		ActivPAL, decrease in break-ups from sedentary for secondary school students, increase in break-ups from sitting in primary school	Verloigne *et al*. (2018)
		Actigraph accelerometer, fourth to fifth grade increased steps (activity level). 7–8th decreased sedentary time	Aspiranti *et al*. (2023)
		Waist-mounted triaxial Accelerometer, fifth grade less sedentary before open learning space, third grade increased their PA in open learning space	Hartikainen *et al*. (2021)
		The GT3X accelerometer failed to detect reductions in sedentary time in comparison with ActivePal	Silva *et al*. (2019)
		ActivPAL monitors (students) and the 25-item Strength and Difficulties questionnaire (teachers), decline in behaviour-related mental health (the students sat down as soon as they could at second observation)	Sherry *et al.* (2020)
Theme 2: Classroom behaviour (definition: active engagement with academic materials and/or following directions of teachers, measured through observation, with or without MTS, real-time classroom actions, and questionnaires)	2.1: On-task behaviour (the time in which the student is focused on the learning)	MTS, prolonged engagement	Erwin *et al*. (2018)
		MTS, improved time on task, fourth to fifth grade	Aspiranti *et al*. (2023)
		FGI (+ Interviews), (students) increased focus	Aminian *et al*. (2015)
		Questionnaire (students), easier to work	Kidokoro *et al.* (2019)
		Interviews (teacher), increased focus, greater concentration	Blake *et al.* (2012)
		Interview (teacher) greater concentration for students that can’t sit for longer periods	Schwenke and Coenen (2022)
		FGI + Interviews, improved classroom behaviour	Clemes *et al*. (2020)
		Questionnaire (students), help to focus with bicycles (half of the group)	Fedewa *et al*. (2021)
		Interview (students), helping with focus, less fidgety	Fedewa *et al*. (2017)
	2.2: Off-task Behaviour (definition: behaviours not related to classroom objectives, rules)	Surveys, distraction on focus and engagement in open learning spaces/when using stability balls	Hulac *et al*. (2022)
		MTS, reduced time on task for seventh to eighth grade	Aspiranti *et al*. (2023)
		Interview (teacher), some rolling around the desks and also reminding the students not to lean on the desk, but not a problem having to remind them	Erwin *et al*. (2018)
		Observation and interviews (teacher + students), moving stations around, moving around, leaning on the desks, and gradually sitting more (at second observation)	Sherry *et al.* (2020)
		Interview (teacher), distraction, as low participation rate (third grade)	Hinckson *et al*. (2013)
		Observation + questionnaire (teacher + students), increase in distraction with bicycles	Fedewa *et al.* (2018)
		Questionnaire (students), increase in distraction with bicycles (half of the group)	Fedewa *et al*. (2021)
		Interview (teacher) students not always compliant during class due to the angle of the bikes	Fedewa *et al*. (2017)
		Questionnaire, relationship with classmates deteriorated in secondary school students	Verloigne *et al*. (2018)
Theme 3: Cognitive performance (definition: assessment through standardized tests, evaluated in controlled settings)	3.1: Positive Cognitive Performance	MAZE probes (reading comprehension), 7–eighth grade	Aspiranti *et al*. (2023)
		Wisconsin Card Sort Test (perseveration and abstract thinking)	Mehta *et al.* (2015)
		FIT (visuospacial abilities, working memory, attention and cognitive flexibility, problem-solving, and executive function)	Parrish *et al*. (2018)
		MAP	Mead and Scibora (2016)
		Flanker Test (selective attention and inhibitory function) third grade and females	Wallace *et al.* (2022)
		Stroop Colour Word Test (inhibition of cognitive interference, abstract reasoning, working memory), improvement in accuracy in the word incongruent stimuli for the IG	Torbeyns *et al.* (2017)
	3.2: Negative cognitive performance	Corsi Block-tapping (visuospatial working memory and short-term memory), group vs. time interaction, both improved but CG better than IG	Silva *et al*. (2022)
		Raven Matrices (general fluid intelligence) group vs. time interaction, CG no change and IG reduced standard score	Silva *et al*. (2022)
		The Print Tool, handwriting quality deteriorated	Metz *et al*. (2020)
		Stroop Colour Word Test, IG slower reaction time than CG	Sherry *et al.* (2020)
Theme 4: Physical and/or mental well-being (definition: a state of health where body and/or mind function at a fulfilling level, subjective perceptions)	4.1: Physical comfort	Modified version of the Nordic Musculoskeletal Questionnaire, musculoskeletal comfort	Ee *et al.* (2018), Aminian *et al*. (2015)
		Seven-item questionnaire, no musculoskeletal discomfort	Sherry *et al.* (2020)
		Nordic musculoskeletal questionnaire, little to no musculoskeletal pain or fatigue	Hinckson *et al*. (2013)
		Interview (teacher), increased body awareness	Schwenke and Coenen (2022)
	4.2 Physical discomfort	11-item questionnaire (on attention, behaviour, and comfort + four open-ended questions), uncomfortable seats	Fedewa *et al*. (2017)
		11-item questionnaire (on academic benefits (subjective perceptions), enjoyment of the bike, and shortcomings of the bike), uncomfortable, too big for the tables and the classroom	Fedewa *et al*. (2021)
		FGI students, bodily tiredness after 4 weeks of standing	Aminian *et al*. (2015)
		Meetings, parents reported children felt tired when standing up, asked to be allowed to sit	Silva *et al*. (2018)
		ActivePAL, usage somewhat distracting	Clemes *et al*. (2020)
	4.3: Emotional satisfaction, enjoyment, and/or self-efficacy	FGI + Interviews, preferred standing workstations	Aminian *et al*. (2015), Schwenke and Coenen (2022), Kidokoro *et al.* (2019)
		The Intervention Rating Profile-15 Survey (teachers)+ 16 items written at a first-grade level (students), positive feedback for learning	Aspiranti *et al*. (2023)
		FGI + Interviews (teachers + students), very positive, liking standing up	Clemes *et al*. (2020)
		FGI + Interviews (students + parents), positive feedback in fourth grade	Hinckson *et al*. (2013)
		FGI + Interviews (teacher/parents/students), happier and more motivated students	Aminian *et al*. (2015), Blake *et al.* (2012), Aspiranti *et al*. (2023), Erwin *et al*. (2018)
		Interview (teacher), lessons more dynamic and motivating for the students, teaching at eye-level improved connection	Schwenke and Coenen (2022)
		Questionnaire (students), self-efficacy + peer reminder of standing up positive	Sudholz *et al*. (2023)
		Questionnaire (students), less sleepiness	Kidokoro *et al.* (2019)
	4.4: Emotional dissatisfaction	Interview (teacher), distracting due to low participation rate in third grade	Hinckson *et al*. (2013)
Theme 5: Challenges (definition: obstacles, practical barriers)	5.1: Physical space	Lack of space to store personal items	Aminian *et al*. (2015), Hinckson *et al*. (2013), Erwin *et al*. (2018)
		Lack of space in classroom	Verloigne *et al*. (2018)
		Bikes take up space	Fedewa *et al*. (2021)
	5.2: Infrastructure	Rotation structure needed, according to teacher and/or students	Schwenke and Coenen (2022)
	5.3: Stations	High cost of stations	Blake *et al.* (2012), Torbeyns *et al.* (2017), Verloigne *et al*. (2018), Wallace *et al.* (2022)
		Cost-effectiveness when using bigger, shared standing stations	Chen *et al.* (2021)
		Not enough stations	Silva *et al*. (2018), Aminian *et al*. (2015), Verloigne *et al*. (2018)
		Desk adjustments necessary as the students grow	Blake *et al.* (2012)
		Bikes noisy	Fedewa *et al*. (2017)

CG, control group; FIT, Figural Intersections Task; IG, intervention group; MAP, Measures of Academic Progress; MTS, momentary time sampling;.

The following sections provide the findings, highlighting key insights from the literature and answering research questions 1 and 2. Research question 3 is addressed through the inductive thematic analysis that follows the summary.

## RESULTS

### Characteristics of classroom-based sedentary behaviour interventions

To address the first research question, this section outlines the core characteristics of research studies investigating classroom design interventions aimed at reducing sedentary behaviour. The section is structured around four key areas: (i) types of classroom design interventions, (ii) age groups and participant characteristics, (iii) geographic distribution of studies, and (iv) research design and methods.

#### Types of classroom design interventions

This subsection describes the different physical design strategies used to reduce sedentary time in classrooms.

Standing desks were the dominant classroom design (Table 1), reflected by 25 studies. These height-adjustable devices allow students to alternate between sitting and standing. The desks are typically paired with stools or chairs, allowing students to stand or sit at their discretion. Cues such as prompts, posters, and reminders from teachers or peers were used to promote standing, though students were not required to remain standing. Additionally, a few studies explored the use of learning spaces equipped with different dynamic furniture designed or open learning spaces to encourage movement, while a few others opted for stability balls as an alternative to traditional chairs to promote active sitting. The feasibility of using bicycles under the desk was studied. The under-the-desk band for fidgeting and the mat under the standing desk were also features studied.

#### Age groups and participant characteristics

Here, the target populations in the studies are outlined with a focus on age distribution and number of participants, given their relevance to assessing the scope and depth of research in this area.

Most articles, 29, focused on children aged 9–12 years, with fewer studies examining younger or older age groups. Sample sizes across the included studies varied widely, ranging from small pilot samples of fewer than 10 participants to larger cohorts exceeding 200 students.

#### Geographic distribution of studies

This part highlights where the research was conducted, identifying international patterns. Most studies were conducted in the USA, followed by several European countries, Australia, and Asia. The overall distribution reflects a concentration on middle childhood across various international contexts, predominantly from the USA.

#### Research design and methods

The methodological approaches used are summarised below, with emphasis on quantitative dominance and study design types.

Quantitative methods constitute the most used framework, employed in 26 studies, with almost half of them being randomized control trials (RCTs), quasi-RCTs with some level of assignment to control, and experimental groups, as well as crossover studies. Slightly below half of these quantitative studies opted for a non-RCT design, including cluster non-RCTs, pre–post studies, and controlled before–after studies. The remaining 34% opted for a mixed-method design primarily consisting of non-RCTs (including cluster non-RCTs), pre–post studies, and controlled before–after studies. No study relied solely on qualitative data. A few studies relied on data sampling that did not involve wearable gears, whereas the rest used an attachable device.

The study examined all collaborations between the research team and end-users to respond to the second research question of to what degree the students are involved in co-designing classrooms to prevent sedentary behaviour during lessons.

### Co-design

None of the included studies explicitly described their intervention as co-designed with students. In fact, no study involved students in the planning or development of interventions in a way that would meet typical definitions of co-design—namely, the collaborative and iterative engagement of end-users throughout the design process.

One study (Silva *et al*. 2018) and its two follow-up analyses (Silva *et al*. 2019, 2022) included some elements that may be interpreted as partial co-design. In these, adjustments to the intervention were made during implementation, based on feedback from parents and teachers. However, these adaptations were not described by the authors as co-design, nor was student involvement mentioned. Given the limited detail and lack of student participation, these studies do not fully meet standard criteria for authentic co-design.

Six other studies (Parrish *et al*. 2018, Verloigne *et al*. 2018, Clemes *et al*. 2020, Aspiranti *et al*. 2023, Sudholz *et al*. 2023, Fedewa *et al*. 2021) collected feedback from students, parents, or teachers, but only after the intervention had concluded. These comments were intended to inform future work and did not influence the design or implementation of the current interventions. Consequently, these instances cannot be considered co-design either, as no iterative or collaborative design process occurred during the study.

### The findings of the thematic analysis

The thematic analysis yielded five main themes, each comprising a set of fourteen nuanced and interrelated sub-themes. The themes and sub-themes from the thematic analysis were: (i) The theme of impact on PA, which entailed tangible effects on the participant caused by physical movement or activity ≥1.5 MET, was identified through accelerometers, which are motion-sensing wearables to determine body position and PA. The sub-themes were PA, changes in posture, and divergent outcomes on physical impact. (ii) The theme of classroom behaviour was understood as the active engagement with academic materials and/or following directions of teachers, measured through observations, with or without momentary time sampling (MTS), and real-time classroom actions. On-task behaviour and off-task behaviour were the sub-themes. (iii) The theme of cognitive performance, which entailed assessment through standardized tests, was evaluated in controlled settings and included positive cognitive performance and negative cognitive performance as sub-themes. (iv) The theme of physical and/or mental well-being, a state of health where the body and/or mind function at a fulfilling level, was assessed by subjective impression. This fourth theme encompassed any changes in physical and/or mental well-being experienced by the students and/or the teacher and/or caregivers (at home) during the intervention. Sub-themes such as physical comfort, physical discomfort, emotional satisfaction, enjoyment, and/or self-efficacy, and emotional dissatisfaction arose. (v) The theme of challenges, which covered obstacles and practical barriers was identified through end-users’ feedback.

Physical space, infrastructure, and stations were the sub-themes.

#### Theme 1: impact on physical activity

*Sub-theme 1.1: increase/decrease in physical activity*

Most studies reported an increase in PA following classroom-based interventions. These increases were observed through objective measures such as accelerometers and heart-rate monitors, with outcomes including higher step counts, greater energy expenditure, and more time spent standing or moving. At the same time, a few studies showed decreased activity levels, notably in interventions using stability balls or under-desk bands.

*Sub-theme 1.2: changes in posture*

Changes in posture were also a consistent finding. Many studies demonstrated a reduction in sedentary time and an increase in standing time, particularly with standing desks and active seating.

*Sub-theme 1.3: divergent outcomes on impact on physical activity*

While many studies demonstrated positive changes, others presented more nuanced or even contradictory results. In some cases, sedentary time increased or gradually returned over time, particularly as the school year progressed. Outcomes also varied depending on student characteristics. Students with higher baseline fitness levels were more likely to break up sitting time and be active while standing. Meanwhile, those with lower initial activity levels—especially girls—tended to benefit the most from the interventions. Age differences were also evident: younger students (Grades 3–5) were generally more active than older peers (Grades 7 and 8 and secondary school students). Some inconsistencies between activity-monitoring devices were noted, such as discrepancies between Actigraph GT3X and ActivPAL in detecting standing time.

#### Theme 2: classroom behaviour

*Sub-theme 2.1: on-task behaviour*

Positive changes in classroom behaviour were found in more than half of the studies. Younger students, in particular, showed improved time-on-task, increased concentration, and enhanced classroom engagement. Students who found it difficult to sit for long periods reported increased focus when given the option to move or stand. In several studies, students said that bicycles and standing desks helped them concentrate, though the effects were not universally positive.

*Sub-theme 2.2: off-task behaviour*

Conversely, off-task behaviour was observed in several studies, often depending on the intervention and the students’ age. Distractions were reported with bicycle desks, especially when participation was inconsistent. Some teachers noted students moving workstations around, leaning excessively on desks, or requiring frequent reminders. In certain contexts, classroom relationships even seemed to suffer, particularly among older students.

#### Theme 3: cognitive performance

*Sub-theme 3.1: positive cognitive performance*

Of the studies assessing cognitive outcomes through standardized testing, several showed improvements in specific areas such as executive function, selective attention, and working memory. Tests like the MAZE, Flanker, Stroop, and Figural Intersections Task revealed performance gains among students using standing desks or other active classroom setups.

*Sub-theme 3.2: negative cognitive performance*

In a few cases, control groups outperformed intervention groups on measures of general fluid intelligence or visuospatial memory. One study also observed a decline in handwriting quality with stability balls. These findings suggest that while movement-friendly interventions may enhance some cognitive functions, others may be unaffected—or even hindered—by changes in posture or classroom setup.

#### Theme 4: physical and/or mental well-being

*Sub-theme 4.1: physical comfort*

Several interventions led to improved physical comfort and reduced fatigue, with students reporting less musculoskeletal discomfort and greater body awareness.

*Sub-theme 4.2: physical discomfort*

Some discomforts were reported, including tiredness after prolonged standing and dissatisfaction with the fit or comfort of bicycle desks.

*Sub-theme 4.3: emotional satisfaction, enjoyment, and/or self-efficacy*

Emotional satisfaction was frequently noted: students and teachers described higher motivation, reduced sleepiness, and a preference for standing or moving throughout the day.

*Sub-theme 4.4: emotional dissatisfaction*

One teacher of younger students (Grade 3) noted that interventions caused distraction when only some students were participating.

#### Theme 5: challenges

*Sub-theme 5.1: physical space*

Implementation challenges were commonly reported. Physical space constraints limited the ability to store personal items and use new equipment effectively.

*Sub-theme 5.2: infrastructure*

Some classrooms required rotation schedules to accommodate all students, creating logistical burdens.

*Sub-theme 5.3: stations*

The cost and quantity of available stations posed additional barriers, particularly for schools unable to afford sufficient equipment. Adjustments for growing children and the noise generated by bicycle desks also emerged as practical concerns.

## DISCUSSION

This scoping review aimed to identify approaches, challenges, and outcomes associated with both classroom design interventions and co-designing with students to create classrooms to reduce sedentary behaviour during lessons.

Most studies (65%) used quantitative methods, with a significant portion employing RCTs and experimental designs, which provide strong causal evidence; however, nearly half used non-RCT design, which may limit causal inferences. A smaller number of studies (34%) used a mixed method, primarily non-RCTs.

In the reviewed studies, classroom design modifications predominantly targeted furniture changes, especially the introduction of standing desks, stability balls, and bicycle desks. Of these, standing desks emerged as the most frequently implemented intervention, likely due to their perceived simplicity and compatibility with traditional classroom layouts. These findings suggest that simple design changes, like introducing standing desks, are both feasible and effective for schools aiming to reduce sedentary time without disrupting instruction. Schools with limited budgets or space might prioritize these scalable changes before adopting more complex interventions.

However, this focus on furniture reflects a narrow conception of classroom design, emphasizing PA over broader environmental or pedagogical transformation. End-user input, particularly from students and teachers, was absent in the selection and integration of these elements, raising questions about the sustainability and contextual fit of such interventions. Moreover, practical challenges, such as limited space for storing personal items, reveal a disconnect between the intervention design and everyday classroom needs (Hinckson *et al*. 2013, Aminian *et al*. 2015, Erwin *et al*. 2018). These findings underscore the need for more holistic and participatory approaches to classroom redesign—ones that go beyond furniture and consider the classroom as a complex, adaptive learning environment.

The other designs also revealed practical challenges. Some furniture-based interventions, such as stability balls and bicycle desks, were reported to interfere with typical classroom tasks due to discomfort, noise, or ergonomic limitations (Fedewa *et al*. 2017, 2021, Metz *et al*. 2020, Hulac *et al*. 2022). These challenges, noted across multiple studies, highlight the need for design solutions that balance movement with functionality.

Concerning the health and behaviour impact in the classroom, the designs using standing desks, stability balls, and bicycles did not seem to have any adverse effects on health outcomes, whether physical or mental well-being, except for some bodily tiredness from standing up (Aminian *et al*. 2015, Silva *et al*. 2018) and inconvenience in using bicycles (Fedewa *et al*. 2017, 2018, 2021). During the interventions, older students (fifth grade, secondary students, and seventh to eighth grade) tended to be more sedentary and spend less time on lecture tasks than their younger peers (Verloigne *et al*. 2018, Hartikainen *et al*. 2021, Aspiranti *et al*. 2023), which may suggest a growing inclination to be seated with increased age, in line with insights drawn from previous research (Kontostoli *et al*. 2021). To achieve long-term and sustainable impact, such interventions may have a more significant impact if started early.

Below, we reflect on how greater student involvement in intervention design might improve relevance and impact.

Co-design was largely absent from the studies included in this review. None of the published intervention employed a co-design methodology (Bowler *et al*. 2021), and no study involved end-users in the design phase. A few studies invited end-users (only teachers and parents) to report and evaluate the progress and were open to feedback and suggestions on the process and the intervention (Silva *et al*. 2018, 2022). The students were only invited to the kick-off presentation of the project and had no further opportunities for direct feedback on the intervention study (Silva *et al*. 2018, 2022). Other studies demonstrated an even lower degree of participation and welcomed input at the end of the intervention, but only for consideration in future studies (Verloigne *et al*. 2018, Clemes *et al*. 2020, Sudholz *et al*. 2023, Fedewa *et al*. 2021). While this may appear to reflect a lack of participatory practise, it is important to acknowledge that many of these studies may not have intended to adopt a co-design framework in the first place. Criticizing them for not doing so risks applying an inappropriate evaluative lens. Instead, it may be more productive to clarify what co-design entails—namely, the collaborative and iterative involvement of end-users in the design, development, and evaluation phases—and to explore how such an approach might influence study outcomes. For instance, co-design could enhance contextual relevance, user engagement, and sustainability of interventions, particularly when working with populations such as students, whose perspectives are often underrepresented.

Therefore, future research could benefit from considering co-design not as a universal requirement, but as a strategic choice aligned with specific goals, contexts, and resource constraints. The absence of direct student involvement in the interventions’ design phase presents a potentially missed opportunity to obtain insights into the sociotechnical elements of the intervention, such as environment, social dynamics in the classroom, and behaviour during lessons for achieving a sustainable change in the norm. This direct student involvement could be accomplished through brainstorming, planning, and decision-making with the students as part of a conscious co-design (Bowler *et al*. 2021, Olsen 2023), with nudging as a complement to motivate the students towards standing and moving (Damgaard and Nielsen 2018, Forberger *et al*. 2019). Participation entails empowerment, a key factor when interacting with end-users (Olsen 2023). When empowered, students are opinion-forming and social individuals who become active agents in changes concerning their lives, in line with Cockerham’s Health Lifestyle Model (Antonovsky 1996) and more recent studies on co-design (Angelöw and Psouni 2025). Co-design also aligns with health promotion (Antonovsky 1996) and increasing health literacy in the rising generation.

Schools and educators can draw from this review to consider low-cost, high-impact changes such as removing traditional chairs part-time, offering flexible furniture layouts, or allocating areas for movement within classrooms. More importantly, involving students in the redesign process—from ideation to implementation—can improve acceptance and use of such environments. This participatory model not only aligns with student-centred pedagogies but can also promote ownership and long-term behavioural change. Policymakers should support schools in piloting co-designed learning spaces and fund research into sustainable movement-friendly classroom models.

### Future research and implications

Future research should explore several avenues for achieving a more significant impact on avoiding sedentary behaviour. First, include especially students in the intervention design process to obtain their voice and input into what the optimal classroom design would be (Holquist *et al*. 2023). Studies ought to investigate how the involvement of students and teachers might change the choices in traditionally selected classroom designs for interventions when tackling the problem of sedentary behaviour. Second, significantly increase the standing time by removing the possibility of returning to a traditional seated position on a chair (so-called nudging) to motivate students towards standing and moving (Damgaard and Nielsen 2018, Forberger *et al*. 2019). This approach of exploratory constraints invites stakeholders to be creative in meeting those goals by setting the problem space or defining certain boundaries, such as abandoning the regular chair. With such a design, focus and outcome measures can be changed from the time spent sitting or standing to behavioural strategies to sustainably shift from sitting to standing during lessons. Third, some movement should be added along with the standing for optimal health outcomes (The Public Health Agency of Sweden 2025). Fourth, alternative qualitative research designs (e.g. observations, interviews, reflexive journals, etc.) could advocate for more nuanced outcomes. The qualitative approach could offer deeper insights into why the shift from sitting to standing and moving is hard and what strategies make the intervention succeed or fail (Higginbottom and Liamputtong 2015). Effective involvement of end-users and more pragmatic research and intervention design would possibly offer better insights into how to sustainably shift from prolonged sedentary behaviour in students, which would positively affect their health outcomes in the future.

### Strengths and limitations

The strength of this study is that it addresses current societal issues with sedentary behaviour, which is a growing problem affecting not only the individual negatively, but in time also the healthcare sector with increasing demands from a less healthy population. This study extends beyond conventional design, looking for a human agency perspective by suggesting co-design for future studies. The limitation of this study is its narrow inclusion criteria. Relevant studies omitting the words intervention or experiment in titles or keywords may not have been included in the study, nor interventions or experiments not written in English. The authors also acknowledge the inherent subjectivity in the interpreting and presenting of the data, which could have influenced the choice of themes and sub-themes in the thematic analysis. Thus, we may be biased and miss potential perspectives relevant to interpreting the data material. To tackle these possible sources of error, we repeatedly reflected on the study’s results and its implications within the entire research team (Lincoln and Guba 1985). Another limitation of this review is the absence of a formal quality appraisal of the included studies. This decision aligns with the methodological guidance for scoping reviews, which aim to map the breadth and nature of the available evidence rather than assess its effectiveness or rigour (Arksey and O'Malley 2005, Tricco *et al*. 2018). However, this may limit the interpretation of the strength of the evidence presented and should be addressed in future systematic or meta-analytic reviews.

## CONCLUSION

This scoping review identified the key elements of interventions aimed at reducing students’ sedentary behaviour in the classroom during lessons. So far, the interventions focused on different types of classroom furniture and allowed students to choose their time of standing at their own discretion, delivering little impact on positive changes in health outcomes, and the interventions were designed without the involvement of end-users.

## Data Availability

The data underlying this review are scientific articles from journals available in databases. These articles are summarized in Table 1.
